# New Terpenoids from *Chamaecyparis formosensis* (Cupressaceae) Leaves with Modulatory Activity on Matrix Metalloproteases 2 and 9

**DOI:** 10.3390/molecules23030604

**Published:** 2018-03-07

**Authors:** Meng-Lun Chang, Hui-Ching Mei, I-Chih Kuo, George Hsiao, Yueh-Hsiung Kuo, Ching-Kuo Lee

**Affiliations:** 1 Graduate Institute of Pharmacognosy, Taipei Medical University, Taipei 11031, Taiwan; chang76819@yahoo.com.tw; 2 Department of Science Education, National Taipei University of Education, Taipei 10671, Taiwan; hcmei@tea.ntue.edu.tw; 3 Department of Microbiology and Immunology, University of British Columbia, Vancouver, BC V6T1Z4, Canada; emgrace970@gmail.com; 4 Graduate Institute of Medical Sciences, Taipei Medical University, Taipei 11031, Taiwan; geohsiao@tmu.edu.tw; 5 Department of Chinese Pharmaceutical Sciences and Chinese Medicine Resources, China Medical University, Taichung 40402, Taiwan; 6 Department of Biotechnology, Asia University, Taichung 41354, Taiwan; 7 School of Pharmacy, Taipei Medical University, Taipei 11031, Taiwan; 8 Ph.D. Program in Biotechnology Research and Development, College of Pharmacy, Taipei Medical University, Taipei 11031, Taiwan

**Keywords:** Cupressaceae, *Chamaecyparis formosensis*, terpenoids, MMP-2, MMP-9, HT-1080

## Abstract

*Chamaecyparis formosensis* is Taiwan’s most representative tree, and has high economic value. To date, only a few active chemical constituents have been reported for *C. formosensis*. In this study, 37 secondary metabolites, including three new compounds (**1**–**3**), were extracted from the leaves of *C. formosensis*. The compounds isolated from the ethyl acetate layer were used at different concentrations to treat HT-1080 human fibrosarcoma cells and to evaluate their effects on matrix metalloprotease 2 (MMP-2) and 9 (MMP-9) expression. Based on extensive analysis of data from high-resolution mass spectrometry (HR-MS) as well as nuclear magnetic resonance (NMR), infrared (IR), and ultraviolet (UV) spectroscopy, the new compounds were identified as 11,12-dihydroxyisodaucenoic acid (**1**), 12-hydroxyisodaucenoic acid (**2**), and 1-oxo-2α,3β-dihydroxytotarol (**3**). Known compounds **4**–**37** were identified by comparing their spectroscopic data with data reported in the literature. Biological activity tests by gelatin zymographic analysis revealed that seven compounds, including new compound **2**, have no cytotoxic effect on HT-1080 cells and were found to increase MMP-2 or MMP-9 expression by 1.25- to 1.59-fold at lower concentrations of 10–50 µM. These naturally derived regulatory compounds could potentially serve as a novel pharmaceutical basis for medical purposes.

## 1. Introduction

The genus *Chamaecyparis* is the main genus of the family Cupressaceae, with five species, *C. formosensis*, *C. lawsoniana*, *C. obtusa*, *C. pisifera*, and *C. thyoides*, and one variety, *C. obtusa* var. *formosana*, spread throughout the world [1]. The genus is native to eastern Asia (Japan and Taiwan), as well as the western and eastern regions of the United States. Besides *C. formosensis* and *C. obtusa* var. *formosana*, the two endemic species that are abundant in Taiwan, a number of studies have reported that the *Chamaecyparis* genus has different types of bioactivity, including antimicrobial, antiinsect, and anticancer activities [2,3,4].

*Chamaecyparis formosensis* is a slow-growing but long-living endemic tree that is usually found at elevations of 1500–2150 m in Taiwan’s central mountains [5]. It is indigenous to the high mountain area and is also known as the Taiwan red cypress because of the reddish-brown color of its bark [6]. The wood is frequently used as lumber and for building, particularly temples and shrines, and for expensive furniture. The plant is known for its strong resistance against wood-decaying fungi and termites [7]. To date, only a few bioactive constituents from the roots, bark, wood, peel, and leaves from this plant have been investigated. [7,8,9,10,11,12,13,14].

Matrix metalloproteinases (MMPs), also known as matrixins, belong to a group of zinc-dependent endopeptidases that break down extracellular matrix (ECM) [15]. Cleaving of collagen, elastin, gelatin, and casein by MMPs [16] is essential for physiological processes such as embryonic development, angiogenesis [17], morphogenesis, reproduction, and tissue resorption and remodeling [18]. MMPs can be subdivided into collagenases (MMP-1, -8, -13), stromelysins (MMP-3, -7, -10), membrane type MMPs (MMP-14, -15, -16), gelatinases (MMP-2, -9), and others (MMP-11, -12). For gelatinases, MMP-2 (gelatinase A) is involved in multiple pathways, including roles in the nervous system, endometrial menstrual breakdown, regulation of vascularization, and metastasis. MMP-9 (gelatinase B), on the other hand, has been associated with numerous somatic illnesses, cardiovascular disorders, tumors [19], and cancers. Moreover, both MMP-2 and MMP-9 take part in the inflammatory, proliferative, and remodeling phases of wound healing [20]. MMP-2 is upregulated during different phases of the wound healing process, whereas MMP-9 expression level is upregulated only during the early phases [21]. The extracellular degradation of collagenase I, III, and IV and elastin due to the increased enzyme activity of MMP-2 and MMP-9 induced by reactive oxygen species results in wrinkle formation and loss of skin elasticity [22].

Until now, little research has examined the relationship between constituents of *C. formosensis* leaves and their bioactivity. Therefore, the aim of this paper is to comprehensively investigate the constituents of leaves from *C. formosensis* and the potential compounds affecting MMP-2 and MMP-9 expression levels.

## 2. Results and Discussion

### 2.1. Compounds Isolated from the Leaves of C. formosensis

**Figure 1:**
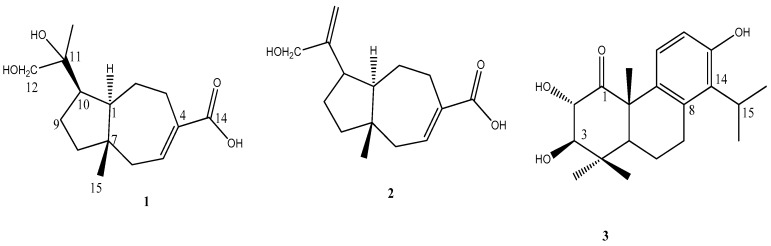
Structures of compounds **1**–**3**.

Methanolic leaf extract of *C. formosensis* was suspended in water and partitioned using ethyl acetate (EA). The EA fraction was subjected to open column chromatography with silica gel to perform gradient elution using an increasing concentration of EA in hexane. MMP-2 and MMP-9 contents were evaluated in cultured HT-1080 human fibrosarcoma cells with compounds screened by the bioassay-guided method: 4(15)-eudesmene-1,6-diol (**4**) [23], dehydrovomifoliol (**5**) [24], pisferal (**6**) [23], pisferol (**7**) [23], pisferic acid (**8**) [25], *O*-methylpisferic acid (**9**) [25], 3β-hydroxysugiol (**10**) [26], 3β-hydroxytotarol (**11**) [26], 1-oxo-3β-hydroxy-13-*O*-methyltotarol (**12**) [23], 1-oxo-3β-hydroxytotarol (**13**) [23], formosanic acid (**14**) [27], pisferanol (**15**) [23], *trans*-communic acid (**16**) [28], phytol (**17**) [29], nortrachelogenin (**18**) [30], 8′β-hydroxynortrachelogenin (**19**) [31], massonianoside A (**20**) [32], cupressoside A (**21**) [23], kaemferol-3-*O*-α-rhamnose (**22**) [33], epicatechin (**23**) [34], catechin (**24**) [33], ametoflavone (**25**) [35], 4′′′-*O*-methylametoflavone (**26**) [36], 4′′′,7-dimethoxyametoflavone (**27**) [37], 7-*O*-methylametoflavone (**28**) [38], hinokiflavone (**29**) [39], 3-(4-hydroxyphenyl)-(2*E*)-2-propenoic acid (**30**) [40], benzoic acid (**31**) [40], 1-(4-hydroxyphenyl)-2,3-dihydroxypropan-1-one (**32**) [41], 27-hydroxyheptacosanoic acid (**33**) [42], methyl 27-hydroxyheptacosanoate (**34**) [42], (2*R*)-(2*R*,4′*R*,8′*R*)-nor-α-tocopherol (**35**) [43], stigmasterol (**36**) [44], and sitostenone (**37**) [44]. All these compounds were identified through 1D- and 2D-NMR, mass spectrometry spectroscopic data analysis, and comparison to the literature. Three previously unreported chemical entities are 11,12-dihydroxyisodaucenoic acid (**1**), 12-hydroxyisodaucenoic acid (**2**), and 1-oxo-2α,3β-dihydroxytotarol (**3**) (Figure 1).

### 2.2. Structural Elucidation of Compounds **1**–**3**

**Figure 2:**
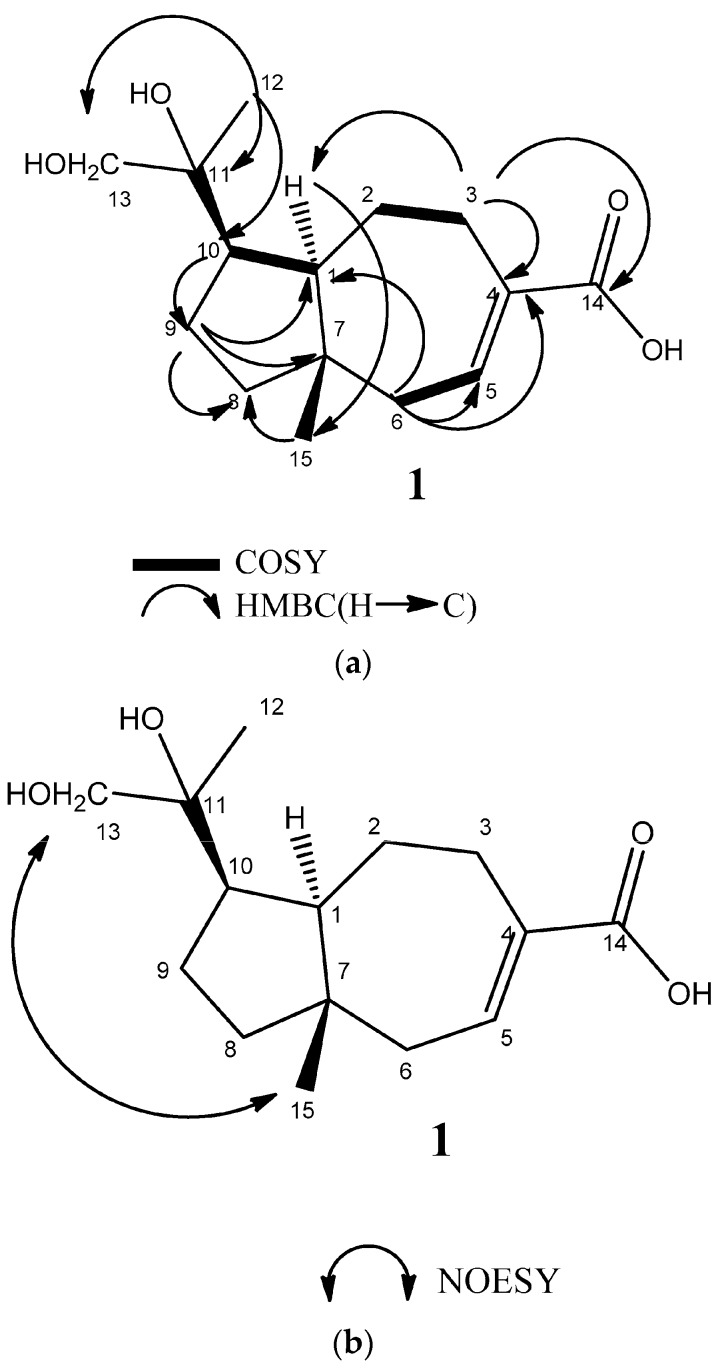
(**a**) Selected HMBC and ^1^H–^1^H COSY spectra of compound **1**; (**b**) selected NOESY spectrum of compound **1**.

Compound **1** was isolated as an amorphous white powder and exhibited an [M − H]^−^ ion peak at *m*/*z* 267.1591(calcd. for C_15_H_23_O_4_, 267.1596) in the negative HRESIMS. This corresponds to a molecular formula of C_15_H_24_O_4_ (four degrees of unsaturation). Its infrared (IR) spectrum showed absorbance of a hydroxyl and conjugated carboxylic acid groups (ν_max_ 3300–2300 and 1690 cm^−1^). The ^1^H-NMR data revealed two methyl groups (δ_H_ 0.80 (s), H-15, and δ_H_ 1.10 (s), H-13), an oxygenated methylene (δ_H_ 3.28 (d, *J* = 10.4 Hz), 3.33 (d, *J* = 10.4 Hz), H-12), and one olefinic proton, δ_H_ 7.10 (m, H-5). The ^13^C NMR data (Table 1) showed 15 resonances, including one conjugated carboxyl carbon (δ_C_ 169.6), two olefinic carbons (δ_C_ 136.4, 142.2), and two oxygenated carbons (δ_C_ 75.9, 70.9). Only two degrees of unsaturation were observed from the ^13^C-NMR data. Accordingly, there were two degrees of unsaturation remaining, which suggested the presence of two rings. The 2D experiments (COSY and HMBC spectra of **1**) (Figure 2a) were used to determine the oxygenated and olefinic functionality of **1**. The HMBC of **1** showed correlations from H-13 (δ_H_ 1.10) to C-10 (δ_C_ 48.6), C-12 (δ_C_ 70.9), and C-11 (δ_C_ 75.9); H-9 (δ_H_ 1.78) to C-1 (δ_C_ 58.0), C-7 (δ_C_ 42.7), and C-8 (δ_C_ 42.4); H-1 (δ_H_ 1.99) to C-15 (δ_C_ 19.7), C-2 (δ_C_ 24.2), C-3 (δ_C_ 30.9), C-8 (δ_C_ 42.4), C-7 (δ_C_ 42.7), and C-11 (δ_C_ 75.9); H-15 (δ_H_ 0.80) to C-8 (δ_C_ 42.4), C-7 (δ_C_ 42.7), C-6 (δ_C_ 43.3), and C-1 (δ_C_ 58.0); H-5 (δ_H_ 7.10) to C-7 (δ_C_ 42.7), and C-14 (δ_C_ 169.6); and H-3 (δ_H_ 3.08) to C-1 (δ_C_ 58.0), olefinic carbons (δ_C_ 136.4, 142.2), and carboxylic acid C-14 (δ_C_ 169.6). In addition, there were ^1^H–^1^H COSY correlations between H-2/H-3 and H-5/H-6. Therefore, carboxylic acid moiety is postulated to be attached to C-4. The NOESY experiment demonstrated the correlations from bridgehead methyl (δ_H_ 0.80, H-15) to methylene hydroxyl group (δ_H_ 3.28, 3.33, H-12) and H-1 (δ_H_ 1.99) to H-10 (δ_H_ 2.59) (Figure 2b), which is an indication that they are in the same phase. Thus, the relative configuration structure of **1** was determined to be 11,12-dihydroxyisodaucenoic acid.

Compound **2** was obtained as an amorphous white powder and assigned the molecular formula C_15_H_22_O_3_ (five degrees of unsaturation) based on its negative HRESIMS. Its IR spectrum showed absorbance of a hydroxyl and α,β-unsaturated carboxylic acid (3300–2300 and 1686 cm^−1^). The ^1^H- and ^13^C-NMR data (Table 1) were very similar to data of a known compound, isodaucenoic acid [45], except the methyl signal (δ_C_ 23.0, δ_H_ 1.69) on C-13 of the isodaucenoic acid was replaced with a methylene signal (δ_C_ 67.7, δ_H_ 3.98, 4.06). The difference suggested that the methyl was replaced by a hydroxymethylene. The relative configuration of **2** was assigned based on the NOESY correlation. The NOESY correlations of H-15/H-12 and H-1/H-10 indicated that they were in the same phase. Thus, the relative configuration of compound **2** is 12-hydroxyisodaucenoic acid.

Compound **3** was isolated as an amorphous white powder with the molecular formula C_20_H_28_O_4_, as determined by negative HRESIMS. It showed an [M − H]^−^ ion at *m*/*z* 331.1903 (calcd. for C_20_H_27_O_4_, 331.1909). Its IR spectrum indicated absorbance of a hydroxyl (3400 cm^−1^), cyclohexanone (1705 cm^−1^), and aromatic groups (1597 cm^−1^).

The ^1^H-NMR spectrum revealed that compound **3** has an isopropyl group attached to a phenyl group (δ_H_ 1.30 (3H, d, *J* = 6.7 Hz), δ_H_ 1.31 (3H, d, *J* = 6.7 Hz), and δ_H_ 3.20 (1H, sept, *J* = 6.7 Hz)), confirming a hydroxyl group located in its *ortho* position, three methyl groups on tertiary carbons (δ_H_ 1.14, 1.15, and 1.61), two *ortho* protons of phenyl (δ_H_ 6.98 (1H, d, *J* = 8.5 Hz) and δ_H_ 6.57 (1H, d, *J* = 8.5 Hz)), and two mutually coupled methine protons at δ_H_ 4.70 (1H, dd, *J* = 10.4, 5.5 Hz) and δ_H_ 3.05 (1H, d, *J* = 10.4 Hz). The ^13^C-NMR data showed 20 resonances, including one carbonyl (δ_C_ 210.4), six aromatic carbons (δ_C_ 153.1, 132.5 (×2), 131.3, 128.9, and 114.4), five methyl carbons (δ_C_ 29.0, 25.3, 20.5, 20.4, and 17.0), two methylene carbons (δ_C_ 29.9 and 18.7), four methine carbons (δ_C_ 85.5, 75.4, 49.8, and 29.6), and two quaternary carbons (δ_C_ 53.5 and 38.6). From the physical evidence, the structure of **3** was classified as a derivative of totarol. ^1^H–^1^H COSY correlation of H-2/H-3 combined with the HMBC correlations from C-1 to H-2, H-20; C-3 to H-2, H-18, H-19; and C-5 to H-19, H-20 indicated that the carbonyl group and the two hydroxyl groups are located at C-1, C-2, and C-3, respectively. The relative configuration of **3** was assigned based on the NOESY correlations of H-20/H-2, H-20/H-19, and H-5/H-3 and the coupling constant. The methine protons of H-2, H-3 appeared at δ_H_ 4.70, 3.05 with a larger coupling constant (*J* = 10.4 Hz), indicating that H-2 and H-3 could be axial protons. Thus, the relative configuration structure of **3** was named 1-oxo-2α,3β-dihydroxytotarol.

### 2.3. Evaluation of Modulatory Effects of Compounds on MMP-2 and MMP-9 Expression in HT-1080 Cells

To obtain the modulatory compounds for MMP-2 and MMP-9 expression, we analyzed the compound-rich EA layer by HPLC. The concentrated extract was subjected to silica gel chromatography. Six fractions were isolated and screened using the bioassay-guided method for their respective MMP activity. Compounds in three fractions with effects of elevating and decreasing MMP-2 expression were further isolated and purified through semi-HPLC techniques.

Thirty-seven purified compounds were classified into eight types of compounds: sesquiterpenoid, diterpenoid, lignan, flavonoid, biflavonoid, aromatic, aliphatic, and steroid compounds. All compounds were individually applied to human fibrosarcoma cell line HT-1080 cells to assess cytotoxicity and MMP-2 and MMP-9 expression. Five out of the eight types, a total of seven compounds, were identified to have modulatory effects on MMP-2 and MMP-9 levels (Table 2).

One of the new sesquiterpenoid compounds had distinct effects on MMP-2 and MMP-9 expression when compared with PMA (a positive control that is known to improve MMP-2 and MMP-9 expression) or EGCG (a negative control that represses MMP-2 and MMP-9 expression). As shown in Table 2, compound **2** elevated MMP-2 expression 1.26-fold (50 µM) and 1.32 fold (10 µM). By observing the structure, we found that the presence of a double bond on C11 of **2** (Figure 1) seemed relevant to the regulation of MMP expression. Little is known about the biological activity of carotene-structured sesquiterpenoids. However, research has been done on Schisanwilsonenes A, a compound structurally similar to **2** (except the carboxyl group on C4 is substituted with a methene alcohol, and a different substituent is attached to C10) with antiviral function [46]. The novel finding of the MMP modulatory effect of carotene type sesquiterpenoid compound **2** can potentially provide insight into the cellular regulatory mechanism. As for the other newly discovered diterpenoid **3**, no changes were detected in MMP-2 or MMP-9 levels.

When analyses were performed on lignan compounds, **18** and **19** had individual effects on MMP-2 and MMP-9 expression (Table 2). Results showed that the hydroxyl groups on C8 or C8′ of lignans could be responsible for regulating the expression of MMP-2 and MMP-9. Flavonoids such as **23** and **24** differ in the stereo position of the hydroxyl group on C3, which resulted in various levels of MMP-2 expression. A related compound with physical significance is epicatechin gallate, which is gallic acid derived from C3 of epicatechin. It is considered to prevent lung cancer metastasis by repressing MMP-2 expression [47,48]. Furthermore, we speculate that the expression of MMP-9 on **26** and **27** is modulated by the number of methyl groups and binding on the C3′-C6″ or C4′-O-C6″ position of a biflavonoid compound. Lee et al. [49] concluded that biflavonoid affects MMP activity and cell viability via binding variation on C2′-C8″ positions, such as C2′-C8” on 2′,8″-biapigenin, C3′-C8″ on sumaflavone, and C3′-C3″ on taiwaniaflavone, corroborating our speculation. All compounds with regulatory effects on MMP expression in this study possess more than 80% viability through HT-1080 MTT cell viability assay. In summary, to investigate the functional groups regulating MMP-2 and MMP-9 expression, we compared the structures of some biologically significant compounds with those of the MMP modulatory compounds in our study. Some distinctive findings show promise to serve as the basis for advanced biochemical examination.

Because of its rarity, *Chamaecyparis formosensis* is understudied and little is known about the species or its composition. However, we discovered several new compounds from leaf extract of *C. formosensis* through bioassay-guided fractionation. These newly identified compounds are highly unique to the species, and we also studied their individual regulatory effect on MMP-2 and MMP-9 levels of HT-1080 human fibrosarcoma cells. Elevated MMP-2 and MMP-9 is thought to induce angiogenesis, inflammation, and cancer metastasis [17], and may also be related to progression of diseases such as hypertension, hyperlipidemia, and diabetes mellitus [50]. On the other hand, MMP-2 and MMP-9 could also be involved in the process of wound healing [20,21]. In this paper, we isolated seven compounds with the ability to upregulate MMP-2 and MMP-9 expression. These naturally derived compounds could serve as candidates to investigate the mechanisms of pathogenesis and wound healing.

## 3. Materials and Methods

### 3.1. General Methods

Optical rotations were measured on a JASCO P-1020 polarimeter. IR spectra were recorded on a JASCO 4100 FT-IR spectrometer. UV spectra were measured on a Helios spectrophotometer (Thermo Scientific, Waltham, MA, USA). NMR data were recorded on a Bruker DRX-500 SB 500 MHz instrument (Bruker, Rheinstetten, Germany) with CDCl_3_ and acetone-d6 as solvent and TMS as internal standard. HRESIMS data were acquired using an LTQ-Orbitrap XL mass spectrometer (Thermo Scientific, Bremen, Germany). Semipreparative HPLC was performed on a Hitachi L-7110 HPLC with a refractive index detector (Thermo Separation Products, Sunnyvale, CA, USA). A Phenomenex Luna silica column (5 μm, 10 × 250 mm, Torrance, CA, USA) and Hibar^®^ Fertigsäule silica column (5 μm, 10 × 250 mm, Merck, Darmstadt, Germany) were used for normal-phase separations. Silica gel (Geduran^®^ Si 60 0.063–0.2 mm, Merck, Darmstadt, Germany) was used for column chromatography. All solvents were either ACS or HPLC grade and were obtained from JT Baker (Phillipsburg, NJ, USA).

### 3.2. Plant Material

Leaves of *Chamaecyparis formosensis* were collected at Cile Hill in Nantou County, Taiwan, in the summer of 2009 and kindly provided to our laboratory by the Taiwan Forestry Research Institute. The sample was authenticated by Dr. Sheng-you Lu (Taiwan Forestry Research Institute), and a voucher specimen (CFL2009-1) was deposited at the School of Pharmacy, Taipei Medical University, Taipei, Taiwan.

### 3.3. Extraction and Isolation

Dried leaves (5.95 kg) of *C. formosensis* were smashed and extracted 3 times with 50 L of methanol, which was then filtered and rotary evaporated to give a brown-black residue (510 g). This residue was then suspended in 3 L of water and partitioned with an equal volume of ethyl acetate 3 times. The ethyl acetate layer was evaporated to dryness under vacuum (200 g). Subsequently, the dried ethyl acetate layer was mixed with 300 g of silica gel (70–230 mesh, Merck), and loaded onto a conditioned open column packed with 2500 g of silica gel and eluted via a stepwise gradient method using mixtures of *n*-hexane, ethyl acetate, and methanol. Five hundred milliliters were collected for each fraction and analyzed using thin-layer chromatography (TLC). TLC was performed on silica gel 60 F254 plates (Merck) using mixtures of *n*-hexane-ethyl acetate for development, and the spots were detected after heating by spraying with vanillin-sulfuric acid. Then, 216 fractions were combined into 6 portions (I–VI) according to the results of the TLC analyses; they were then redissolved in a minimum volume of *n*-hexane/ethyl acetate mixtures for subsequent HPLC analysis.

Portion I eluted by *n*-hexane/ethyl acetate (90:10) was purified by semipreparative HPLC (Hibar^®^ Fertigsäule, 10 × 250 mm) using *n*-hexane/ethyl acetate (95:5) as the eluent at a flow rate of 3 mL/min to yield compounds **6** (4.1 mg, tR = 7.0 min), **9** (3.9 mg, tR = 10.2 min), and **16** (3.3 mg, tR = 15.1 min). Portion II was eluted by *n*-hexane/ethyl acetate (80:20) and purified by semipreparative HPLC (Phenomenex^®^ Luna, 10 × 250 mm) using *n*-hexane/acetone (90:10) as the eluent at a flow rate of 3 mL/min to yield **4** (4.7 mg, tR = 31.8 min), **15** (4.2 mg, tR = 16.0 min), **17** (5.2 mg, tR = 25.2 min), **35** (3.1 mg, tR = 35.7 min), **36** (3.4 mg, tR = 21.0 min), and **37** (3.1 mg, tR = 27.0 min). The same portion was purified by semipreparative HPLC (Phenomenex^®^ Luna, 10 × 250 mm) using *n*-hexane/ethyl acetate (85:15) as the eluent at a flow rate of 3 mL/min to separate **7** (5.7 mg, tR = 26.7 min), **8** (3.5 mg, tR = 17.6 min), **11** (3.3 mg, tR = 27.6 min), **12** (2.7 mg, tR = 41.3 min), **14** (4.1 mg, tR = 19.3 min), **31** (2.7 mg, tR = 20.4 min), and **33** (3.6 mg, tR = 34.5 min). Portion III, eluted by *n*-hexane/ethyl acetate (60:40), was purified by semipreparative HPLC (Phenomenex^®^ Luna, 10 × 250 mm) using *n*-hexane/ethyl acetate (60:40) as the eluent at a flow rate of 3 mL/min to yield **19** (11.5 mg, tR = 26.0 min), **2** (4.5 mg, tR = 24.4 min), **3** (3.6 mg, tR = 21.7 min), **5** (435 mg, tR = 38.4 min), **13** (2.6 mg, tR = 22.1 min), **30** (3.2 mg, tR = 42.5 min), and **34** (3.8 mg, tR = 40.1 min). Portion IV, eluted by *n*-hexane/ethyl acetate (40:60), was purified by semipreparative HPLC (Hibar^®^ Fertigsäule, 10 × 250 mm) using *n*-hexane/ethyl acetate 40:60 as the eluent at a flow rate of 3 mL/min to yield compounds **27** (11.2 mg, tR = 13.0 min) and **10** (4.2 mg, tR = 14.6 min). The same portion was purified by semipreparative HPLC (Phenomenex^®^ Luna, 10 × 250 mm) using *n*-hexane/ethyl acetate (50:50) as the eluent at a flow rate of 3 mL/min to yield **18** (8.3 mg, tR = 14.7 min), **23** (5.5 mg, tR = 23.0 min), and **32** (4.7 mg, tR = 70.3 min). The same portion was purified by semipreparative HPLC (Hibar^®^ Fertigsäule, 10 × 250 mm) using *n*-hexane/ethyl acetate (30:70) as the eluent at a flow rate of 3 mL/min to yield **1** (6.3 mg, tR = 27.1 min), **24** (3.2 mg, tR = 16.4 min), and **25** (8.0 mg, tR = 19.0 min). Portion V, eluted by *n*-hexane/ethyl acetate (30:70), was purified by semipreparative HPLC (Hibar^®^ Fertigsäule, 10 × 250 mm) using *n*-hexane/ethyl acetate (50:50) as the eluent at a flow rate of 3 mL/min to yield compounds **26** (7.8 mg, tR = 15.0 min), **28** (5.6 mg, tR = 16.5 min), and **29** (3.4 mg, tR = 7.6 min). Portion VI was eluted by 100% CH_3_OH and purified by semipreparative HPLC (RP Hibar^®^ Fertigsäule, 10 × 250 mm) using CH_3_CN/H_2_O (25:75) as the eluent at a flow rate of 3 mL/min to yield **20** (4.3 mg, tR = 13.2 min), **21** (5.2 mg, tR = 21.5 min), and **22** (3.6 mg, tR = 30.2 min).

### 3.4. 11,12-Dihydroxyisodaucenoic Acid (**1**)

Amorphous white powder; ${\left[\alpha \right]}_{D}^{25}$ + 16.1 (c 0.1, CH_3_OH); UV λ_max_ MeOH nm: 262 (log*ε* 1.6), 325 (log*ε* 0.5); IR (neat) ν_max_ 3300–2300, 2923, 2857, 1690, 1457, 1150; negative ESIMS *m*/*z* 267.1591 [M − H]^−^ (calcd. for C_15_H_23_O_4_, 267.1596); for ^1^H and ^13^C NMR data, see Table 1.

### 3.5. 12-Hydroxyisodaucenoic Acid (**2**)

White powder; ${\left[\alpha \right]}_{D}^{25}$ + 13.5 (c 0.10, CH_3_OH); UV λ_max_ MeOH nm: 263 nm (log*ε* 1.5), 320 nm (log*ε* 1.0); IR (neat): 3300–2300, 1686, 1630, 1274; negative HRESIMS *m*/*z* 249.1494 [M − H]^−^ (calcd. for C_15_H_21_O_3_, 249.1491); for ^1^H- and ^13^C-NMR data, see Table 1.

### 3.6. 1-Oxo-2α,3β-dihydroxytotarol (**3**)

White powder; [α]${\left[\alpha \right]}_{D}^{25}$ + 17.0 (c 0.10, CH_3_OH); UV λ_max_ MeOH nm: 260 nm (log*ε* 1.8), 320 nm (log*ε* 1.2); IR (neat) ν_max_ 3400, 2927, 2854, 1705, 1597, 1455, 1281, 1093; negative HRESIMS *m*/*z* 331.1903 [M − H]^−^ (calcd. for C_20_H_27_O_4_, 331.1909); for ^1^H- and ^13^C-NMR data, see Table 3.

### 3.7. Cell Culture

HT1080 human fibrosarcoma cells were purchased from American Type Culture Collection (ATCC: CCL-121) and cultured in RPMI-1640 medium (Gibco), which was supplemented with 10% heat-inactivated fetal bovine serum, 100 U mL^−1^ penicillin, and 100 mg mL^−1^ streptomycin. The cell cultures were maintained in a humidified incubator at 37 °C in 5% CO_2_/95% air.

### 3.8. Gelatin Zymography

Gelatin zymography was used to determine expression and activities of MMP-2 and MMP-9 [16]. HT1080 cells were seeded in 24-well plates using serum-free medium for 24 h cell adhesion and growth. After giving the cells some time for cell adhesion and growth, they were treated with various concentrations (10 µM, 50 µM, and 100 µM) of compounds **1**–**37** for 48 h before the MMP-2 and MMP-9 activities were analyzed. The medium was collected for data analysis.

### 3.9. Statistical Analysis

The experimental results are expressed as mean ± SEM. Data were analyzed using one-way ANOVA and subsequently Student–Newman–Keuls test with *p* < 0.05, *p* < 0.01, and *p* < 0.001 to examine statistical significance.

## Figures and Tables

**Table 1:** ^1^H- and ^13^C-NMR spectral data of **1** and **2** (acetone-*d*_6_, 500 MHz).

Position	1		2	
	δ_H_	δ_C_	δ_H_	δ_C_
1	1.99 (1H, m)	58.0	1.89 (1H, m)	57.2
2	1.48 (1H, m) 2.54 (1H, m)	24.2	1.25 (1H, m) 1.54 (1H, m)	24.0
3	1.80 (1H, m) 3.08 (1H, m)	30.9	3.03 (1H, m) 1.90 (1H, m)	28.4
4		136.4		134.5
5	7.10 (1H, m)	142.2	7.20 (1H, m)	144.8
6	2.40 (1H, m) 2.10 (1H, m)	43.3	2.45 (1H, dd, *J* = 14.0, 9.6) 2.03 (1H, m)	43.0
7		42.7		42.9
8	1.38 (1H, m) 1.48 (1H, m)	42.4	1.58 (1H, br dd, *J* = 11.6, 6.7) 1.46 (1H, br dd, *J* = 11.6, 6.7)	42.5
9	1.65 (1H, m) 1.78 (1H, m)	27.7	1.70 (2H, m)	30.1
10	2.59 (1H, m)	48.6	2.91 (1H, m)	45.6
11		75.9		151.7
12	3.28 (1H, d, *J* = 10.4 Hz) 3.33 (1H, d, *J* = 10.4 Hz)	70.9	4.06 (1H, d, *J* = 14) 3.98 (1H, d, *J* = 14)	67.7
13	1.10 (3H, s)	22.0	4.93 (1H, br s), 5.16 (1H, br s)	111.4
14		169.6		172.8
15	0.80 (3H, s)	19.7	0.81 (3H, s, CH_3_)	19.9

**Table 2:** Compounds that modulate matrix metalloprotease 2 (MMP-2) and MMP-9 expression to various degrees.

Compound	MMP-2	MMP-9
PMA		2.5 * (Pro MMP-9 **, 1 μM ***)
EGCG	0.2–0.4 (Pro MMP-2, 100 μM)	
13-Hydroxyisodaucenoic acid (**2**)	1.26 (50 µM) 1.32 (10 µM)	
Nortrachelogenin (**18**)	1.30 (50 µM) 1.59 (10 µM)	1.54 (10 µM)
8′β-hydroxynortrachelogenin (**19**)	1.23 (50 µM) 1.34 (10 µM)	
Epicatechin (**23**)	1.31 (50 µM) 1.40 (10 µM)	
Catechin (**24**)	1.29 (50 µM) 1.17 (10 µM)	
4′′′-*O*-methylametoflavone (**26**)		1.42 (Pro MMP-9 ***, 10 µM)
4′′′,7-dimethoxyametoflavone (**27**)		1.18 (Pro-MMP-9, 50 µM)

* Number of folds by which MMP-2 expression was elevated compared to the control group. ** Increased form of MMP expression. ***Concentrations of compounds used to treat HT-1080 cells.

**Table 3:** ^1^H- and ^13^C- NMR spectral data of **3** (CDCl_3_, 500 MHz).

Position	δ_H_ (mult., *J* in Hz)	δ_C_
1		210.4
2	4.70 (1H, dd, *J =* 10.4, 5.5 Hz)	75.4
3	3.05 (1H, d, *J* = 10.4 Hz)	85.5
4		38.6
5	1.45 (1H, dd, *J* = 12.1, 1.2 Hz)	49.8
6	1.55 (1H, m)	18.7
	2.02 (1H, m)	
7	2.67 (1H, m)	29.9
	2.95 (1H, m)	
8		132.5
9		131.3
10		53.5
11	6.98 (1H, d, *J* = 8.5 Hz)	128.9
12	6.57 (1H, d, *J* = 8.5 Hz)	114.4
13		153.1
14		132.5
15	3.20 (1H, sept)	29.6
16	1.30 (3H, d, *J* = 6.7 Hz)	20.5
17	1.31 (3H, d, *J* = 6.7 Hz)	20.4
18	1.14 (3H, s)	29.0
19	1.15 (3H, s)	17.0
20	1.61 (3H, s)	25.3
